# Antimicrobial stewardship programs in a network of Canadian acute care hospitals: a cross-sectional survey

**DOI:** 10.1017/ash.2025.181

**Published:** 2025-05-29

**Authors:** Erin McGill, Andrew Neitzel, Jessica J Bartoszko, Maureen Buchanan-Chell, Jennifer Grant, Jenine Leal, Stephanie Smith, Reena Titoria, Olivia Varsaneux, Charles Frenette

**Affiliations:** 1 Public Health Agency of Canada, Ottawa, ON, Canada; 2 Alberta Health Services, Edmonton, AB, Canada; 3 British Columbia Centre for Disease Control, Vancouver, BC, Canada; 4 Provincial Health Services Authority, Vancouver, BC, Canada; 5 McGill University Health Centre, Montreal, QC, Canada

## Abstract

**Objective:**

Antibiotics are essential to combating infections; however, misuse and overuse has contributed to antimicrobial resistance (AMR). Antimicrobial stewardship programs (ASPs) are a strategy to combat AMR and are mandatory in Canadian hospitals for accreditation. The Canadian Nosocomial Infection Surveillance Program (CNISP) sought to capture a snapshot of ASP practices within the network of Canadian acute care hospitals. Objectives of the survey were to describe the status, practices, and process indicators of ASPs across acute care hospitals participating in CNISP.

**Design:**

The survey explored the following items related to ASP programs: 1) program structure and leadership, 2) human, technical and financial resources allocated, 3) inventory of interventions carried and implemented, 4) tracking antimicrobial use; and 5) educational and promotional components.

**Methods:**

CNISP developed a 34-item survey in both English and French. The survey was administered to 109 participating CNISP hospitals from June to August 2024, responses were analyzed descriptively.

**Results:**

Ninety-seven percent (106/109) of CNISP hospitals responded to the survey. Eighty-four percent (89/106) reported having a formal ASP in place at the time of the study. Ninety percent (80/89) of acute care hospitals with an ASP performed prospective audit and feedback for antibiotic agents and 85% (76/89) had formal surveillance of quantitative antimicrobial use. Additionally, just over 80% (74/89) provided education to their prescribers and other healthcare staff.

**Conclusions:**

CNISP acute care hospitals employ multiple key aspects of ASP including implementing interventions and monitoring/tracking antimicrobial use. There were acute care hospitals without an ASP, highlighting areas for investigation and improvement.

## Introduction

Antimicrobials have been a great addition to modern medicine, drastically improving our ability to combat life-threatening infections and allowing immune-suppressing therapies that would previously have carried a high risk of fatal infections.^1^ However, the misuse and overuse of these agents has led to unintended consequences, notably the rise of antimicrobial resistance (AMR), which has become a significant public health concern.^2^ This inappropriate use restricts the effectiveness of available treatments for various infections and poses a growing challenge to patient care, complicating efforts to manage infections.^3,4^

In response, antimicrobial stewardship programs (ASPs) have emerged as a critical strategy to combat AMR in healthcare settings. They involve a systematic approach to guiding healthcare professionals on the judicious use of antimicrobials. Their goal is to optimize the selection, dosing, duration, and administration routes of antimicrobial agents in effort to ensure their effectiveness while minimizing unnecessary exposure.^5^ ASPs are crucial for combatting AMR, enabling coordinated interventions that measure and improve antimicrobial use (AMU), thus ensuring optimal patient outcomes.^6^

Research has consistently shown the effectiveness of ASPs in enhancing appropriate antibiotic use, improving clinical outcomes, and reducing adverse events, including AMR.^7,8^ Moreover, ASPs contribute to cost savings by decreasing the financial burden associated with unnecessary antibiotic treatments.^9,10^ Despite the clear benefits, the implementation of ASPs remains inconsistent across the globe. According to a 2021 World Health Organization (WHO) survey, only 33% of countries had established guidelines and practices for antimicrobial stewardship.^11^ While there is no Government of Canada mandate for ASPs, Accreditation Canada requires all acute care institutions to have an ASP to meet accreditation requirements.^12,13^ Accreditation is voluntary but it does provide formal recognition by peers, both within the hospital and across the country and a way to assess and improve accountability in healthcare delivery. Accreditation Canada provides benchmarking over time and compared to other peer comparators, which can help identify strengths and opportunities for improvement.^14^

In Canada, the Canadian Nosocomial Infection Surveillance Program (CNISP) plays a pivotal role in monitoring healthcare-associated infections (HAIs) and AMR. CNISP is a collaboration between the Public Health Agency of Canada (PHAC), the National Microbiology Laboratory (NML), the Association of Medical Microbiology and Infectious Disease Canada, and over 100 sentinel acute care hospitals across the country. CNISP’s activities include surveillance of HAIs, including viral respiratory infections, antimicrobial-resistant organisms such as methicillin-resistant *Staphylococcus aureus* (MRSA), vancomycin-resistant *Enterococcus* (VRE), carbapenemase-producing organisms (CPOs), and AMU. The CNISP network is representative and includes hospitals from all ten provinces and one territory and represents approximately 37% of acute care beds across Canada.^15^

In 2024, CNISP conducted a cross-sectional survey to understand the scope of ASP practices in acute care hospitals across its network. The objective of this survey was to provide an overview of the strategies, practices, and process indicators associated with ASPs in Canadian acute care hospitals.

## Methods

The Consensus-Based Checklist for Reporting of Survey Studies (CROSS) was used to guide the reporting of this cross-sectional survey.^16^

### Survey development

A 34-item online survey was created and consisted of radio buttons, checklists and open-ended questions. The survey was reviewed by a working group of subject matter experts including infection control professionals, infectious disease doctors, pharmacists, and epidemiologists.

The survey questions were adapted from the Centers for Disease Control and Prevention’s Core Elements of Hospital Antibiotic Stewardship Programs Assessment Tool and a published survey from the Netherlands allowing the results to be compared to other countries’ programs.^17,18^ Questions were developed with the goal of capturing the seven core elements of ASPs: leadership commitment, accountability, pharmacy expertise, action, tracking, reporting and education. The survey explored the following items: (1) program structure, leadership and team composition; (2) human, technical and financial resources allocated; (3) inventory of specific targeted interventions carried out and level of implementation; (4) methods of tracking of antimicrobial use; and (5) education and promotional activities. The final survey was piloted by members of the CNISP team and an infectious disease physician to ensure usability and to measure how much time it would take for completion. The full survey is available in Supplemental Materials; all questions were mandatory.

### Survey participants and administration

Hospitals eligible to participate were Canadian acute care hospitals that were participating in CNISP in June 2024. Under the CNISP Terms of Reference, there is opportunity to participate in special projects, e.g., surveys, where there is financial compensation to incentivize participation. A secure web-based platform called LimeSurvey was used to administer the survey via e-mail to participating CNISP hospitals.^19^ The survey was available from June 1^st^ to August 30^th^, 2024. Respondents had the ability to save their responses as a draft and return to the survey later. Survey responses reflect antimicrobial stewardship programs during this period. Reminder e-mails were sent in July and August to increase participation. The questionnaire was distributed to the entire CNISP network, consisting of 109 hospitals. Surveys were distributed to the hospital’s main point of contact, and it was recommended that the person leading the hospital’s ASP complete the survey.

For reporting purposes, CNISP groups hospital networks into three geographic regions: East (New Brunswick, Newfoundland and Labrador, Nova Scotia, and Prince Edward Island), Central (Ontario, Quebec, and Nunavut), and West (Alberta, British Columbia, Manitoba, Saskatchewan).

### Data entry and analysis

Electronic survey respondents entered their information directly into LimeSurvey. The unit of analysis was the hospital. Survey results were exported from LimeSurvey and uploaded into R studio version 4.3.2^20^ for analysis. Duplicate responses were excluded based on hospital identifiers, as were responses from hospitals that opened the survey but did not respond to any questions and/or complete their submission. A descriptive analysis of responses including proportions, mean, median, and range was conducted to examine ASP practices at a national level.

### Privacy and ethical considerations

Individual hospital responses were aggregated, and the ASP practices of individual hospitals are not reported. The survey was considered exempt from the requirement for ethics approval or approved by the research ethics boards at CNISP hospitals if required by institution-specific policies.^21^

## Results

### Overview

A total of 72 responses were received, representing 97.2% (106/109) of CNISP participating acute care hospitals. Three hospitals in the CNISP network did not complete the survey. Certain hospitals belong to a network where a single response from a staff member may represent multiple hospitals. The median hospital bed size was 177 beds (range 2–1105) and approximately half (57/106) of hospitals were teaching hospitals (Table 1). Over half of the hospitals that responded to the survey were adult hospitals (60/106, 56.6%). However, there was excellent representation of pediatric tertiary care centers in the survey, as 12 of the 13 (92.3%) pediatric tertiary care centers participating in CNISP responded to the survey.

**Table 1.** Characteristics of Canadian Nosocomial Infection Surveillance Program (CNISP) hospitals that responded to the antimicrobial stewardship program survey, June 1 to August 30, 2024

Characteristic	Responses (n = 106)	CNISP hospitals (n = 109, %)
**Geographic distribution**		
East	26	26 (100%)
Central	36	39 (92.3%)
West	44	44 (100%)
**Hospital Beds**		
Range (IQR)	2 to 1,105 (72, 417)	2 to 1,1105 (73, 417)
Mean	268	270
Median	177	178
**Hospital Size**		
Small (1–200 beds)	55	56 (98.2%)
Medium (201–499 beds)	33	34 (97.1%)
Large (500+ beds)	18	19 (94.7%)
**Hospital Type**		
Adult	60	60 (100%)
Mixed	34	35 (97.1%)
Pediatric	12	13 (92.3%)
**Teaching Hospital**		
Teaching	56	59 (94.9%)
Non-teaching	50	50 (100%)

Note*. The proportion indicated is representation of survey responses to actual CNISP hospital characteristics.*

Of the 106 sites that completed the ASP survey, 84.0% (89/106) acute care hospitals indicated that they have an ASP. The other 16.0% (17/106) sites indicated that they did not have an ASP; these were primarily small sites (<200 beds) (15/17 (88.2%)). The year in which sites formalized their ASP ranged from 2001 to 2022, median 2014. All results described in this paper refer to the CNISP acute care hospitals that indicated they have an ASP.

Of the 89 acute care hospitals indicating they have an ASP, only 19 (21.3%) met all seven core elements of an ASP (hospital leadership commitment, accountability, pharmacy expertise, action, tracking, reporting, and education). These were primarily adult hospitals (10), followed by mixed (6) and pediatric (3). Majority were located in the Western region of Canada (14/19) and were evenly distributed across hospital size (7 large (500+ beds), 7 medium (201–499 beds), and 5 small (1–200 beds)).

All hospitals with an ASP program had a pharmacist on the ASP team and 90.0% had an infectious disease (ID) physician and/or infection control specialist physician (Table 2). Seventy-six percent of sites reported having a microbiologist as part of the ASP team (Table 2). Teaching hospitals were more likely to have an ID specialist than non-teaching hospitals (52/54, 96.3% vs 28/35, 80.0% (*P* = 0.03), respectively). Five hospitals did not report a medical doctor (MD) on their ASP committee, these were primarily mixed sites (n = 4) and of medium size (n = 4).

**Table 2.** Composition of the antimicrobial stewardship program team/committee at Canadian Nosocomial Infection Surveillance Program (CNISP) participating hospitals

Team composition	N = 89 *^1^*
Pharmacist	89 (100%)
MD: Infectious diseases or infection control specialist	80 (90.0%)
Microbiologist	68 (76.4%)
Infection control professional	60 (67.4%)
Other MD specialists (e.g., Intensivist, respirologist, internist, family medicine, hemato-oncologist, emergency or surgeon)	40 (44.9%)
Data analyst	34 (38.2%)
Hospital Director	20 (22.4%)
Quality department	19 (21.3%)
Director of professional services	12 (13.5%)
Council of physicians, dentists and pharmacists	2 (2.2%)

1 n (%).

### Hospital leadership commitment and accountability, and resources

Ninety-three percent (83/89) of acute care hospitals participating in CNISP with an ASP had leadership that provided ASP pharmacists dedicated time to manage their programs and conduct daily stewardship interventions.

There were differences in the number of funded FTE positions compared to what hospitals reported as the FTE support for ASP (Table 3). The reported number of FTE pharmacists was slightly higher than the reported funded positions (Table 3). However, the reported number of FTE MDs was lower than funded positions (Table 3). Reported vs funded positions for IT support staff and analytical support were very similar (Table 3).

**Table 3.** Comparison of reported funded vs actual full-time equivalents (FTEs) for different positions

FTE positions	Mean, median (range) FTE
**Pharmacist**	
Funded	1.05, 1.0 (0–3.7)
Actual	1.13, 1.0 (0–5)
**MD**	
Funded	0.36, 0.2 (0–4)
Actual	0.24, 0.2 (0.05–1)
**IT support staff**	
Funded	0.15, 0.09 (0–0.5)
Actual	0.16, 0.09 (0–0.5)
**Analytical support staff**	
Funded	0.23, 0.09 (0–1)
Actual	0.22, 0.09 (0–1)

**Table 4.** List of antimicrobials for which Canadian Nosocomial Infection Surveillance Program acute care hospitals performed prospective audit and feedback

Antimicrobials	N = 80^1^
Carbapenems^2^	41 (51.3%)
Daptomycin^3^	36 (45.0%)
Piperacillin/tazobactam^2^	31 (38.8%)
Fluoroquinolones^2^	27 (33.8%)
Tigecycline^3^	20 (25.0%)
Ceftolozane/tazobactam^3^	17 (21.3%)
Ceftazidime/avibactam^3^	9 (11.3%)
Imipemen/relebactam^3^	8 (10.0%)
Fidaxomicin^2^	8 (10.0%)
Fourth generation cephalosporin^2^	7 (8.8%)
Isavuconazole	6 (7.5%)
Amphotericin B	5 (6.3%)
Echinocandins	5 (6.3%)
Fosfomycin^3^	3 (3.8%)
Foscarnet	2 (2.5%)

Note: antifungals do not have an AWaRe classification.

1 (%);

2 AWaRe classification = Watch;

3 AWaRe classification = Reserve.

Sixty-two percent (55/89) of hospitals indicated that the hospital board of directors provided a budget for their ASP. Approximately half (43/89 (48.3%)) of ASP programs regularly scheduled meetings, with an average of 5.5 meetings a year (range 1–12 per year).

### Action

**Figure 1.**
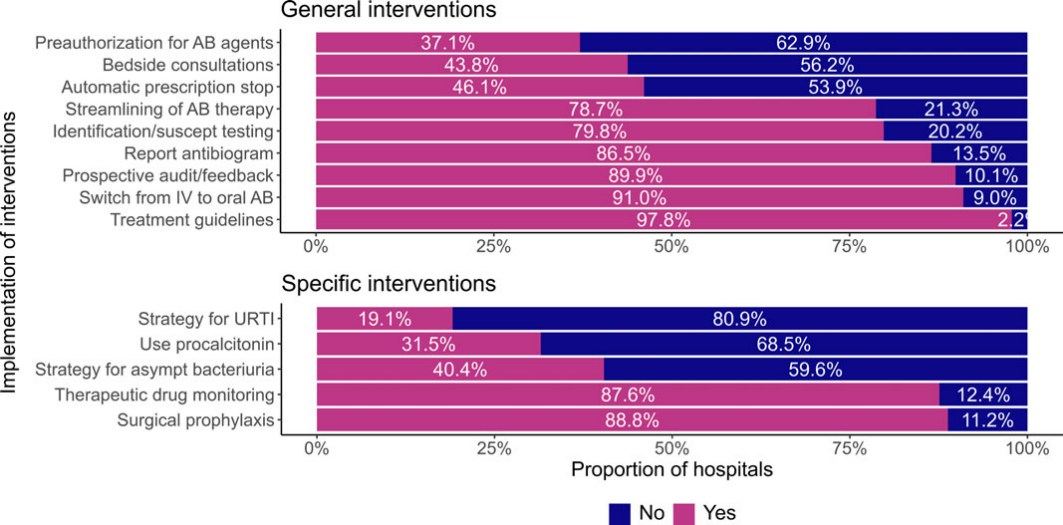
Proportion of Canadian Nosocomial Infection Surveillance Program acute care hospitals implementing general antimicrobial stewardship program (ASP)interventions (above) and specific ASP interventions (below). *Note*: AB = antibiotic; suscept = susceptibility; UTRI = upper respiratory tract infection; asymp = asymptomatic; IV = intravenous.

Almost all (97.8%) of CNISP acute care hospitals with an ASP have treatment guidelines and a large proportion (80/89 (89.9%)) performed prospective audit and feedback for antimicrobials (Figure 1). Most (69/80 (86.3%)) hospitals indicated they conducted prospective audit and feedback several (>1) times a week. Thirty-six percent (29/80) conducted prospective audit and feedback on all hospital units and 63.8% (51/80) performed it on targeted units. The most common targeted units were internal medicine (37/51 (72.5%)), surgical unit (35/51 (68.6%)), and adult intensive care unit (ICU) (29/51 (56.9%)).

Approximately half (39/80 (48.8%)) of CNISP acute care hospitals with an ASP performed audit/feedback for all antimicrobials. When targeted antimicrobials were recorded, the most common antimicrobials reviewed were carbapenems (41/80 (51.3%), daptomycin (36/80 (45.0%)) and piperacillin/tazobactam (31/80 (38.8%)) (Table 4). Only 37.1% (33/89) of acute care hospitals with an ASP performed preauthorization for specific antibiotic agents (Figure 1).

Regarding specific interventions employed at CNISP hospitals with an ASP, most had implemented interventions for surgical prophylaxis (79/89 (88.8%)), followed by therapeutic drug monitoring and optimal dose management (78/89 (87.6%)) and a strategy for decreasing use of antibiotics in asymptomatic bacteriuria (36/89 (40.4%)) (Figure 1). Fewer sites (17/89 (19.1%)) had implemented a strategy for upper respiratory tract viral infections (Figure 1).

### Tracking

**Figure 2.**
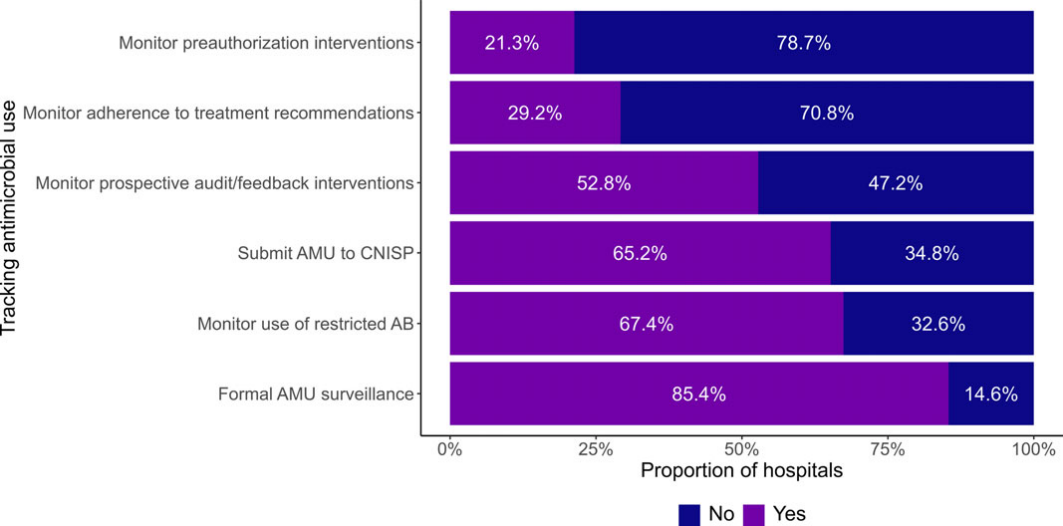
Proportion of Canadian Nosocomial Infection Surveillance Program acute care hospitals employing different methods of tracking antimicrobial use.

Eighty-five percent (76/89) of CNISP sites with an ASP have formal surveillance of quantitative antimicrobial use (Figure 2). Different indicators were used in AMU surveillance: 86.8% (66/76) used defined daily doses, 64.5% (49/76) used days of therapy, 42.1% (32/76) used expenditure/antibiotic dollars and 9.2% (7/76) used length of stay, grams/quantity of antimicrobials or exposed patients. Eighty percent (61/76) of CNISP sites use quantitative software, whereas 18.4% (14/76) of CNISP sites manually tracked this information. One site did not answer the question on software.

Sixty-seven percent (60/89) of sites with an ASP monitored the use of restricted antibiotic agents (Figure 2). The most common method for monitoring use of restricted antibiotic use was computerized alerts when prescribing restricted antibiotics (32/89 (36.0%)), followed by verifying if diagnostic tests were performed (29/89 (32.6%)). Less common but still used to monitor use of restricted antibiotic use was electronic order forms with a decision tree at 21.3% (19/89). Only 13.5% (12/89) of sites used an antibiotic order form to help monitor use of restricted antibiotics.

### Education and reporting

The majority (74/89 (83.1%)) of ASPs provided education to prescribers and other relevant staff on optimal prescribing, adverse reactions from antibiotics, and antibiotic resistance. Sixty-seven percent (60/89) of ASPs provided education to prescribers as part of the prospective audit and feedback process with most sites using prospective audit and feedback (58/60 (96.7%)), followed by grand rounds (44/60 (73.3%)) as the methods of education.

As part of education regarding issues that may not require antibiotic use, 71.9% (64/89) of respondents had addressed asymptomatic bacteriuria, 68.5% (61/89) had addressed prolonged surgical prophylaxis and 19.1% (17/89) had addressed upper respiratory tract viral infections.

Ninety-one percent (81/89) of sites produced an annual antibiogram (cumulative antibiotic susceptibility) report and 67.4% (60/89) had distributed the current antibiogram (i.e., for the 2023 calendar year) to prescribers.

## Discussion

The CNISP national survey, developed in collaboration with an expert working group, provides a critical benchmark for assessing ASP practices across a targeted group of Canadian hospitals. The survey offers valuable insights on ASPs that can guide future improvements, inform policy development, and foster collaboration across institutions.

At the time of the study, the majority of CNISP hospitals had a formalized ASP in place (84.0% (89/106)). As of 2013, Accreditation Canada mandates that hospitals must have an ASP in place to maintain accreditation standards.^12,13^ Although the year of implementation was missing for some hospitals, 42/83 (50.6%) had an ASP before it was mandatory. It is concerning that 17 sites still reported not having an ASP in 2024 and do not meet Accreditation Canada standards. Given the global threat AMR poses, perhaps policymakers/professional societies should make ASPs mandatory, regardless of accreditation status. In 2021, according to the WHO, 33% of hospitals worldwide had implemented an ASP.^11^

Only 19/89 (21.3%) of CNISP hospitals with an ASP met all seven core elements of an ASP. These findings suggest a potential area for improvement for Canadian acute care hospitals to consider including addition core elements to their ASP to improve their response to AMR.

Our analysis revealed that pharmacists, physicians, infectious disease specialists, and microbiologists were key members of ASPs within the CNISP network. This aligns with several reports,^18,22,23^ which emphasized the central role of these professionals in ASPs. We observed that non-teaching hospitals were less likely to include an infectious disease specialist compared to teaching hospitals. This disparity was consistent with findings by Kallen et al. who also reported a correlation between hospital teaching status and the presence of infection control specialists in Dutch hospitals.^18^ Teaching hospitals, typically affiliated with universities, are often located in urban areas where ID specialists are more concentrated.^24^ Due to their urban location and university ties, teaching hospitals are perhaps more likely to employ ID specialists. Approximately 50% of CNISP hospitals are teaching hospitals, whereas data from the Canadian Institute for Health Information indicate that teaching hospitals represent about 10% of all hospitals in Canada.^25^

Prospective audit and feedback are central to ASP strategy and the Society for Healthcare Epidemiology of America (SHEA) Clinical Practice Guidelines for Implementing an Antibiotic Stewardship Program strongly recommends preauthorization and/or prospective audit and feedback.^25^ Results from the survey showed that 89.9% of CNISP acute care hospitals with an ASP performed prospective audit and feedback for antibiotic agents.

Monitoring and tracking antibiotic prescribing, the impact of interventions and important outcomes are crucial to identify opportunities for improvement.^17^ It is important for hospitals to monitor and benchmark AMU by formal surveillance of AMU. Eighty-five percent of CNISP acute care hospitals with an ASP conducted formal surveillance of AMU. As a comparison, the US CDC reports that standardized AMU surveillance occurs in nearly half of NHSN hospitals.^22^

Studies have shown that preauthorization has been associated with a significant reduction in the use of restricted agents.^25,26,28^ Sixty-eight percent of CNISP hospitals monitored the use of restricted antibiotic agents and the most common method used was computerized alerts when prescribing restricted antibiotics. Bahar *et al.* also noted that the implementation of computerized decision support systems influenced the use of antibiotics by reducing their consumption.^29^ Decision trees and machine learning algorithms have also been shown to be useful tools in reducing broad-spectrum antibiotic use.^30,31^ Currently only 22% of CNISP hospitals use electronic forms with decision trees to assist with prescribing antibiotics, potentially highlighting an area for improvement.

Education is a fundamental component of every ASP, playing a critical role in its success and long-term sustainability.^17^ Within the CNISP network, over 80% of hospitals with ASPs provide education to prescribers and other relevant staff, with prospective audit and feedback being the most commonly employed method. This aligns with Howard *et al*., who found that 89% of hospitals worldwide incorporated education for healthcare staff as part of their ASP.^24^

Reporting is also crucial to an ASP, as it is important for hospitals to monitor and benchmark their antibiotic use and provide routine updates to prescribers, pharmacists, nurses, and leadership on process and outcome measures.^17^ The US CDC reported that 43% of hospitals were reporting their antibiotic use to the NHSN in 2022, which is less than the 65.2% of CNISP acute care hospitals reporting their AMU data to CNISP.^22^ However, a direct comparison is difficult to make as CNISP is a sentinel hospital network and NHSN is a national network.

### Strengths

This study had several strengths. The survey achieved a very high response rate of 97.2%, limiting non-response selection bias. Responses were from hospitals that varied in region, hospital size and type, teaching status, and is reflective of current ASP practices within the CNISP network which represents 37% of Canadian acute care beds.^15^ This study provides helpful information in describing ASP programs in Canadian acute care hospitals.

### Limitations

CNISP is a sentinel network and therefore the results of the survey may not be generalizable to all hospitals across Canada. Sites that indicated their ASP was regional would also share their ASP team/committee members with other acute care hospitals in the region and FTE results may be an estimation for certain sites. The survey was conducted at a single point in time so changes over time in hospitals’ ASPs cannot be assessed. Cause and effect are also unable to be assessed, as the study was cross-sectional and the implementation of an ASP is not contrasted with AMR use and outcomes at CNISP hospitals. Adherence to ASP policies was also not measured.

## Conclusion

The CNISP national survey provides valuable insights into the state of ASPs across a subset of Canadian hospitals, highlighting both strengths and areas for improvement. With a high response rate and diverse representation, the survey provides helpful information on ASP practices and offers a foundation for tracking future progress. CNISP acute care hospitals employ multiple key aspects of ASP including implementing interventions and monitoring/tracking antimicrobial use. Furthermore, there is evidence that CNISP ASP practices and experience aligns with other countries. CNISP hospitals that reported no ASP were smaller (<200 beds) which could imply resource constraints, but still means they fall short of Accreditation Canada standards.

Future Canadian ASP studies should consider exploring ASP practices at non-CNISP hospitals, evaluating the effectiveness of ASPs in improving antimicrobial use in Canada and adherence to reported ASP policies.

## Supporting information

Mcgill et al. supplementary material Mcgill et al. supplementary material

## Supplementary material

To view supplementary material for this article, please visit https://doi.org/10.1017/ash.2025.181
