# Supplementary material for: Antimicrobial stewardship programs in a network of Canadian acute care hospitals: a cross-sectional survey
Source: Antimicrob Steward Healthc Epidemiol. 2025 May 29;5(1):e122. doi: 10.1017/ash.2025.181 (PMC12122392; doi:10.1017/ash.2025.181)
Supplement: Mcgill et al. supplementary material [file S2732494X25001810sup001.docx]

# Appendix I: CNISP ASP Survey

**Acute-Care Hospital Antimicrobial Stewardship Program Survey**

Task Authorization for the Canadian Nosocomial Infection Surveillance Program

This survey aims to describe the status, strategies, and process indicators of Antimicrobial Stewardship Programs (ASPs) across acute-care hospitals participating in the Canadian Nosocomial Infection Surveillance Program (CNISP). ASPs are a coordinated hospital program that promote the appropriate use of antimicrobials by healthcare professionals to reduce the emergence of resistance. They involve appropriate selection, dosing, route, and duration of antimicrobial therapy, which help preserve the future effectiveness of antimicrobials by reducing their misuse and overuse (1). There are a wide variety of techniques that can be implemented in ASPs and studies on their effectiveness vary as well (1-5). For example, a prospective cluster-randomized trial showed that the technique of prospective audit and feedback was effective in reducing antibiotic use within adult inpatient populations (364.9 days per 1000 patient days in intervention group vs 384.2 days per 1000 patient days in control group) (2). Both the World Health Organization and Public Health Agency of Canada have emphasized antimicrobial stewardship in their action plans to combat antimicrobial resistance (6,7).

This survey was adapted from the Centers for Disease Control and Prevention’s Core Elements of Hospital Antibiotic Stewardship Programs Assessment Tool and a published survey from the Netherlands (8,9). It should take approximately 20 minutes to complete. In the online survey platform, you can save and resume your work as many times as needed. **We strongly recommend the survey be completed by the person that leads your hospital’s ASP team/committee. If you do not have an ASP team/committee, please indicate ‘No’ in the first question. Please complete one survey per hospital (i.e., CHEC number)**. Responses to this survey should reflect current hospital-level policies and will be kept confidential. Your hospital will not be identified in any reports that are generated, including those that might be submitted for publication.

If you have any questions, please contact us via email at: [cnisp-pcsin@phac-aspc.gc.ca](mailto:cnisp-pcsin@phac-aspc.gc.ca).

1. **What is the name of your hospital? ____________**

1. **Do you currently have a formal Antimicrobial Stewardship Program (ASP) for this hospital?**

 Yes, we currently have a formal ASP program

 No

**IF NO,** end of survey.

**IF YES,** please proceed to the questions below.

Team composition

1. **What year was your ASP formalized? __________**

1. **Please specify who is on the ASP team/committee (*select all that apply*):**

 MD: Infectious diseases or infection control specialist

 Other MD specialists: Intensivist, respirologist, internist, family medicine, hemato-oncologist, emergency or surgeon

 Pharmacist

If there is a pharmacist on the ASP team/committee, do they have formal antimicrobial stewardship training?

 Yes – MAD ID, SIDP, etc.

 Yes – specialized fellowship/residency

 Yes – local self-training with microbiologist/infectious disease specialist/pharmacist

 No

 Microbiologist

 Hospital Director

 Director of professional services

 Quality department

 Council of Physicians, Dentists and Pharmacists

 Infection control professional

 Data analyst

 Other, please specify: _________________________

1. **How many times (on average) does the ASP team/committee meet in a calendar year? ___________**

1. **To whom does your ASP team/committee formally report? S*elect all that apply*.**

 Province/territory

 Council of Physicians, Dentists and Pharmacists

 Department of Professional Services

 Hospital CEO

 Other, please specify: _________

1. **Does your ASP team/committee have a written program adopted by your administration describing mandates, objectives, operational structure, and reporting?**

 Yes

 No

Hospital Leadership Commitment and Accountability, and Resources

1. **Does hospital leadership provide stewardship program lead pharmacist(s) dedicated time to manage the program and conduct daily stewardship interventions?**

 Yes

 No

**If yes, provide the total number of funded ASP-associated full-time equivalent (FTE) lead pharmacists: __________**

**If yes, provide the total number of actual FTEs spent by ASP-associated lead pharmacists on ASP activities: __________**

1. **Does hospital leadership provide stewardship program lead MD(s) dedicated time to manage the program and conduct daily stewardship interventions?**

 Yes

 No

**If yes, provide the total number of funded ASP-associated FTE lead MDs: __________**

**If yes, provide the total number of actual FTEs spent by ASP-associated lead MDs on ASP activities: __________**

1. **Does hospital leadership provide stewardship program leader(s) with IT support to effectively operate the program?**

 Yes

 No

**If yes, provide the total number of funded ASP-associated FTE IT support staff: __________**

**If yes, provide the total number of actual FTEs spent by ASP-associated IT support staff on ASP activities: __________**

1. **Does hospital leadership provide stewardship program leader(s) with analytical support to effectively operate the program?**

 Yes

 No

**If yes, provide the total number of funded ASP-associated FTE analytical support staff: __________**

**If yes, provide the total number of actual FTEs spent by ASP-associated analytical support staff on ASP activities: __________**

1. **Do you have clerical support for your program (executive assistant, administrative technician)?**

 Yes

 No

**If yes, provide the total number of funded ASP-associated FTE clerical support staff: __________**

**If yes, provide the total number of actual FTEs spent by ASP-associated clerical support staff on ASP activities: __________**

1. **Does the Hospital Board of Directors provide a budget for the ASP?**

 Yes

 No

1. **Do stewardship program leader(s) have regularly scheduled meetings with facility leadership and/or the hospital board to report and discuss stewardship activities, resources, and outcomes?**

 Yes

 No

**If yes, how many times (on average) do they meet in a calendar year? ___________**

Action: Implementation of interventions

1. **Does your facility perform prospective audit and feedback for antibiotic agents?**

Prospective audit and feedback broadly refers to reviewing and discussing a patient’s antimicrobial therapy with the prescriber after the antimicrobial was prescribed, which if done in real time, may or may not lead to a change in therapy.

 Yes

 No

**If yes, on which hospital units?**

 All hospital units

 Targeted hospital units, please select all that apply from the list below:

 Neonatal ICU

 Pediatric ICU

 Adult ICU

 ER

 Internal medicine unit

 Surgical unit

 Hematology/Oncology unit

 General pediatric unit

 Long-term care unit

 Other, please specify: _________

**If yes, for which antibiotics?**

 All antibiotics

 Carbapenems,

 All carbapenems

 Select, please specify:____________

 Imipemen/relabactam

 Fluoroquinolones

 All fluoroquinolones

 Select, please specify:____________

 Daptomycin

 Tigecycline

 Fidaxomycin

 Ceftazidime/avibactam

 Ceftolozane/tazobactam

 Piperacillin/tazobactam

 Fourth generation cephalosporin, please specify:_________

 Fosfomycin

 Isavuconazole

 Amphotericin B

 All forms

 Select, please specify:____________

 Echinocandins, please specify: ____________

 Foscarnet

 Other, please specify:

________________________________________________

________________________________________________

________________________________________________

________________________________________________

**If yes, how often?**

 Once per week

 2-3 times per week

 4-5 times per week

 On a continuous basis

**If yes, how is your individual feedback provided? S*elect all that apply*.**

 Verbal direct

 Written in chart

 Email

 Text

 Electronic chart alert

 Other, please specify: _________

1. **Does your facility perform preauthorization for specific antibiotic agents?**

Preauthorization broadly refers to prescribing restricted agents only after authorisation.

 Yes

 No

**If yes, for which antibiotics does your facility perform preauthorization?**

Please specify: ___________

1. **Does your facility have facility-specific treatment guidelines to assist with antibiotic selection for common clinical conditions?**

 Yes

 No

**If yes, for which common clinical conditions? S*elect all that apply*.**

 Urinary tract infections

 Respiratory tract infections and pneumonias

 Sepsis

 Skin and soft tissue infections

 Surgical prophylaxis

 Febrile neutropenia

 Other: ____________

1. **Has your hospital implemented an intervention or strategy for:**

Switch from intravenous to oral antibiotic therapy

 Yes

 No

Streamlining / de-escalation of empirical antibiotic therapy

 Yes

 No

Therapeutic drug monitoring and optimal dose management by pharmacists (e.g., by weight, creatinine clearance)

 Yes

 No

Automatic antibiotic prescription stops

 Yes

If yes, after how many days? Please specify: __________

 No

Surgical prophylaxis (e.g., standardized protocol, pre-printed orders)

 Yes

 No

1. **Does your hospital perform mandatory bedside consultations when prescribing restricted agents?**

Restricted antibiotic use may be limited to certain indications, prescribers, services, patient populations, or combinations thereof.

 Yes

 No

1. **Does your microbiology laboratory report antibiogram in cascade (i.e., only shows results of certain antibiotics in presence of resistance)**

 Yes

 No

1. **Does your hospital use procalcitonin to manage antibiotic use?**

 Yes

 No

1. **Does your laboratory perform rapid identification and susceptibility tests?**

 Yes

 No

1. **Does your laboratory have a strategy to decrease use of antibiotics in asymptomatic bacteriuria?**

 Yes

 No

Tracking Antimicrobial Use and Outcomes

1. **Do you currently have formal surveillance of quantitative antimicrobial use (AMU) for this hospital?**

 Yes, we currently have formal surveillance of quantitative antimicrobial use

 No

**If yes, what year was your antimicrobial use surveillance program formalized? __________**

**If yes, please specify what indicators your program uses (*select all that apply*):**

 Defined daily doses

 Days of therapy

 Length of therapy

 Expenditure/antibiotic dollars (budget spent)

 Grams/quantity of antimicrobials

 Exposed patients

 Other, please specify: ___________________

**If yes, please specify the software quantitative use data is collected by *(select all that apply):***

 Centricity /GE

 SyPhaC / Logibec

 GesphaRx /CGSI

 Msys / Quadramed

 Antibiotics / Nosotech

 APSS-Data/ Lumed

 Manually

 Other, please specify: ________________

1. **Does your ASP track antibiotic use by submitting AMU data to CNISP?**

 Yes

 No

**If no, why not? _______________**

**If yes, do you find CNISP’s site-specific reports useful?**

 Yes, please specify:_________

 No, please specify:_________

**If yes, do you use CNISP’s site-specific reports to guide and/or implement ASP interventions?**

 Yes

 No

**If yes, does your ASP share CNISP’s site-specific reports on AMU with prescribers?**

 Yes

 No

1. **How does your ASP monitor the use of restricted antibiotic agents? Please complete the following:**

Restricted antibiotic use may be limited to certain indications, prescribers, services, patient populations, or combinations thereof.

**Do you have an antibiotic order form?**

 Yes

 No

**Do you have computerized alerts when prescribing restricted antibiotic agents?**

 Yes

 No

**Do you have an electronic order form with decision tree to help MDs prescribe antibiotics?**

 Yes

 No

**Do you check whether diagnostic tests were performed when prescribing restricted antibiotic agents?**

 Yes

 No

1. **Does your ASP monitor prospective audit and feedback interventions by tracking acceptance of the feedback or recommendations?**

 Yes

 No

1. **Does your ASP monitor preauthorization interventions by tracking which agents are being requested for which conditions?**

 Yes

 No

1. **Does your ASP monitor adherence to facility-specific treatment recommendations?**

 Yes

 No

**If yes, for which treatment recommendations? S*elect all that apply*.**

 Urinary tract infections

 Respiratory tract infections and pneumonias

 Sepsis

 Skin and soft tissue infections

 Surgical prophylaxis

 Febrile neutropenia

 Other: ____________

1. **Is an annual antibiogram (cumulative antibiotic susceptibility report) produced for this hospital?**

 Yes

 No

1. **Has your facility distributed a current antibiogram (i.e. for the 2023 calendar year) to prescribers?**

 Yes

 No

Education

1. **Does your ASP provide education to prescribers and other relevant staff on optimal prescribing, adverse reactions from antibiotics, and antibiotic resistance?**

 Yes

 No

1. **Does your ASP provide education to prescribers as part of the prospective audit and feedback process (sometimes called “handshake stewardship”)?**

 Yes

 No

**If yes, what does the education look like? *Select all that apply*.**

 Teaching at service rounds

 Written service reports

 Grand rounds

 Personal annual feedback

 Handshake stewardship

1. **Of the issues below that do not require antibiotic use, which has your institution specifically addressed? S*elect all that apply.***

 Asymptomatic bacteriuria

 Upper respiratory tract viral infections

 Prolonged surgical prophylaxis

 None of the above

Thank you for participating in this survey!

**References**

1. Core Elements of Hospital Antibiotic Stewardship Programs | Antibiotic Use | CDC [Internet]. [cited 2024 Jan 2]. Available from: <https://www.cdc.gov/antibiotic-use/core-elements/hospital.html>
2. Chen JZ, Hoang HL, Yaskina M, Kabbani D, Doucette KE, Smith SW, et al. Efficacy and safety of antimicrobial stewardship prospective audit and feedback in patients hospitalised with COVID-19 (COVASP): a pragmatic, cluster-randomised, non-inferiority trial. Lancet Infect Dis [Internet]. 2023 Jun 1 [cited 2024 Jan 2];23(6):673. Available from: /pmc/articles/PMC9977404/
 3. Molina J, Peñalva G, Gil-Navarro M V., Praena J, Lepe JA, Pérez-Moreno MA, et al. Long-Term Impact of an Educational Antimicrobial Stewardship Program on Hospital-Acquired Candidemia and Multidrug-Resistant Bloodstream Infections: A Quasi-Experimental Study of Interrupted Time-Series Analysis. Clinical Infectious Diseases. 2017 Dec 15;65(12):1992–9.
4. Lawes T, Lopez-Lozano JM, Nebot CA, Macartney G, Subbarao-Sharma R, Dare CRJ, et al. Effects of national antibiotic stewardship and infection control strategies on hospital-associated and community-associated meticillin-resistant Staphylococcus aureus infections across a region of Scotland: a non-linear time-series study. Lancet Infect Dis [Internet]. 2015 Dec 1 [cited 2024 Jan 2];15(12):1438–49. Available from: <https://pubmed.ncbi.nlm.nih.gov/26411518/>

5. Zay Ya K, Win PTN, Bielicki J, Lambiris M, Fink G. Association Between Antimicrobial Stewardship Programs and Antibiotic Use Globally: A Systematic Review and Meta-Analysis. JAMA Netw Open [Internet]. 2023 Feb 9 [cited 2024 Jan 2];6(2):E2253806. Available from: <https://pubmed.ncbi.nlm.nih.gov/36757700/>
6. Public Health Agency of Canada. Pan-Canadian Action Plan on Antimicrobial Resistance [Internet]. [cited 2024 Jan 2]. Available from: <https://www.canada.ca/content/dam/phac-aspc/documents/services/publications/drugs-health-products/pan-canadian-action-plan-antimicrobial-resistance/pan-canadian-action-plan-antimicrobial-resistance.pdf>
7. WHO Library Cataloguing-in-Publication Data Global Action Plan on Antimicrobial Resistance [Internet]. 2015. Available from: [www.paprika-annecy.com](file:///C:\Users\reena.titoria\AppData\Local\Microsoft\Windows\INetCache\Content.Outlook\17WB193O\www.paprika-annecy.com)

8. The Core Elements of Hospital Antibiotic Stewardship Programs ANTIBIOTIC STEWARDSHIP PROGRAM ASSESSMENT TOOL.
 9. Kallen MC, Ten Oever J, Prins JM, Kullberg BJ, Schouten JA, Hulscher MEJL. A survey on antimicrobial stewardship prerequisites, objectives and improvement strategies: Systematic development and nationwide assessment in Dutch acute care hospitals. Journal of Antimicrobial Chemotherapy. 2018 Dec 1;73(12):3496–504.
